# Key Role for the Organic Anion Transporters, OAT1 and OAT3, in the *in vivo* Handling of Uremic Toxins and Solutes

**DOI:** 10.1038/s41598-017-04949-2

**Published:** 2017-07-10

**Authors:** Wei Wu, Kevin T. Bush, Sanjay K. Nigam

**Affiliations:** 1 Department of Medicine, University of California, San Diego, 9500 Gilman Drive, La Jolla, CA 92093 USA; 2 Department of Pediatrics, University of California, San Diego, 9500 Gilman Drive, La Jolla, CA 92093 USA; 3 Departments of Medicine, Pediatrics, and Cellular and Molecular Medicine, University of California, San Diego, 9500 Gilman Drive, La Jolla, CA 92093 USA

## Abstract

***In vitro*** data indicates that the kidney proximal tubule (PT) transporters of uremic toxins and solutes (e.g., indoxyl sulfate, p-cresol sulfate, kynurenine, creatinine, urate) include two “drug” transporters of the organic anion transporter (OAT) family: OAT1 (SLC22A6, originally NKT) and OAT3 (SLC22A8). Here, we have examined new and prior metabolomics data from the *Oat1KO* and *Oat3KO*, as well as newly obtained metabolomics data from a “chemical double” knockout (*Oat3KO* plus probenecid). This gives a picture of the *in vivo* roles of OAT1 and OAT3 in the regulation of the uremic solutes and supports the centrality of these “drug” transporters in independently and synergistically regulating uremic metabolism. We demonstrate a key ***in vivo*** role for OAT1 and/or OAT3 in the handling of over 35 uremic toxins and solutes, including those derived from the gut microbiome (e.g., CMPF, phenylsulfate, indole-3-acetic acid). Although it is not clear whether trimethylamine-N-oxide (TMAO) is directly transported, the *Oat3KO* had elevated plasma levels of TMAO, which is associated with cardiovascular morbidity in chronic kidney disease (CKD). As described in the Remote Sensing and Signaling (RSS) Hypothesis, many of these molecules are involved in interorgan and interorganismal communication, suggesting that uremia is, at least in part, a disorder of RSS.

## Introduction

Uremic toxins/retention solutes comprise a large set of molecules that are normally cleared and excreted by the kidney, but which accumulate in the plasma of patients with renal insufficiency^1^. The accumulation of these endogenous compounds in the plasma (or failure in their excretion) can lead to the manifestation of uremic syndrome affecting the functions of other organs which, if untreated in its final stages, can be fatal^1^.

Uremic toxins have been divided into the following subgroups of compounds: (1) small water-soluble, non-protein-bound (e.g., urea); (2) small protein-bound (e.g., indoxyl sulfate); and (3) middle molecules (mainly small and large peptides)^1, 2^. Among these, the importance of water-soluble and protein-bound toxins has received considerable attention over the past several years^1^. Many of these are small, charged molecules whose uptake from the plasma and excretion via the kidney is mediated by transport proteins expressed on proximal tubule (PT) cells^3–5^. Among these are the multispecific transporters, organic anion transporter 1 (OAT1, SLC22A6, originally NKT^6^) and its close homolog OAT3 (SLC22A8)^7^, which are abundantly expressed on the basolateral membrane of PT cells (as well as several other epithelial tissues) and which together represent two of the most important transporters involved in the renal uptake and excretion of a wide variety of drugs, exogenous toxins, nutrients, and endogenous metabolites, including uremic toxins^5, 8, 9^.

OAT1 and OAT3 have previously been shown to interact with many of these uremic toxins and solutes *in vitro* and considerable *in vivo* data indicate that the OATs also handle many endogenous metabolites, including a number of uremic toxins and/or retention solutes^5, 8–10^. For example, previous targeted and untargeted limited metabolomics analyses of the *Oat1KO* and *Oat3KO* animals, albeit limited in that some were performed with older methods, instruments and databases, revealed significant alterations (up in plasma, down in urine) in the concentration of several uremic toxins and/or uremic retention solutes, including indoxyl sulfate, kynurenine, creatinine and urate^11–13^. Since it is well-established that both the *Oat1KO* and *Oat3KO* animals display the expected defects in the handling of drugs and toxins^12, 14–22^, such alterations in the plasma and/or urine concentrations of endogenous metabolites in the *Oat1KO* and *Oat3KO* animals are expected to reflect changes in their handling due to the absence of the transporters. Here, using a more comprehensive metabolomics analysis, levels of over 90 reported uremic toxins and uremic retention solutes were analyzed from the plasma of *Oat1KO* and *Oat3KO* animals, as well as from *Oat3KO* mice treated with probenecid, an inhibitor of OAT-mediated transport (“chemical double knockouts”)^23^.

## Results

Despite clearly functioning as drug transporters, considerable *in vitro* and *in vivo* data indicate that OAT1 and OAT3 also handle many endogenous metabolites, including a number of uremic toxins and/or retention solutes^5, 8–10, 24^. In this study, levels of uremic toxins and uremic solutes were analyzed from the plasma of the *Oat1KO* and *Oat3KO*, as well as from *Oat3KO*s treated with probenecid (“chemical double knockouts”). Among the more than 600 metabolites examined, ~90 of them are water-soluble or protein-bound uremic toxins/retentions solutes identified by a number of groups (Supplemental Table S1)^1, 2, 25–29^. Partial least squares discriminant analysis of these uremic toxins/retention solutes revealed separation of both *Oat1KO* and *Oat3KO* groups from their wildtype controls (Supplemental Figure 1).

While adding a few molecules which accumulate in the plasma of the *Oat1KO* (e.g., cysteine, kynurenate and S-adenosylhomocysteine), the metabolomics analysis of the plasma from the *Oat1KO* largely agreed with previous studies (Table 1)^12, 13^. For example, kynurenine, methionine and orotate, whose concentration was found to be altered in previous metabolomics analyses^12, 13^, also accumulated in the plasma of the *Oat1KO*s studied here (Table 1). On the other hand, analysis of the *Oat3KO* revealed considerably more alterations than previous attempts^30^, including significant increases in the plasma concentration of numerous uremic toxins/retention solutes, among them trimethylamine N-oxide (TMAO), indoxyl sulfate, p-cresol sulfate and 3-carboxy-4-methyl-5-propyl-2-furanpropanoate (CMPF) (Table 1). Taken together with *in vitro* data (Supplemental Table S2), the new *in vivo* metabolomics data showing the plasma accumulation of a number of uremic toxins provide strong support for the notion that *Oat1* and *Oat3* independently play an important role in the uptake and handling of a wide variety of water soluble and protein-bound uremic toxins/retention solutes^12, 13, 30, 31^. Moreover, certain compounds (e.g., kynurenine, indolelactate, indoxyl sulfate, methionine, creatinine, p-cresol sulfate and putrescine) appear to be *in vivo* substrates for both OATs.

**Table 1:** Uremic toxins/retention solutes with significantly altered concentrations in *Oat1* and *Oat3* knockout mice based on previous and current metabolomics studies. ^a^OAT1 references for previous studies: PMID:16354673; PMID:2146605). ^b^OAT3 references for prior studies (PMID:18270321; PMID:18508962; PMID:2238083; PMID:2390220). ^c^The number in parentheses is the fold increase in plasma concentration for the listed metabolite. *p ≤ 0.05 for all fold increases except those for SAH and CMPF (p<0.1).

**Previous Studies**		**Additional Metabolites Found in This Study**	
**OAT1** ^**a**^	**OAT3** ^**b**^	**OAT1** ^**c**^	**OAT3** ^**c**^
N2,N2-Dimethylguanosine	Creatinine	Cysteine (2.3)	1-Methyl-imidazoleacetate (1.5)
N-Methyladenosine	Urate	Kynurenate (1.6)	2-Aminophenol sulfate (3.2)
Indolelactate		SAH (2.6)*	2-Oxindole-3-acetate (3.9)
Indoxyl sulfate			CMPF (2.6)*
Kynurenine			N-Acetyltryptophan (2.7)
Methionine			Catechol sulfate (3.9)
Orotate			Citrulline (1.3)
Phenylsulfate			Imidazole propionate (4.5)
p-hydroxy-phenyllactic acid			Indoleacetate (2.8)
Urate			Indolelactate (4.6)
Xanthurenate			Indoxyl sulfate (3.8)
			Mannitol/sorbitol (1.7)
			p-Cresol sulfate (3.8)
			Phenylsulfate (2.8)
			TMAO (4.5)

However, the most significant increases in plasma concentration of uremic toxins were observed in the *Oat3KO*s treated with probenecid—a so-called “chemical double knockout” (Tables 2 and 3). Thus far it has not been possible to create an OAT1/OAT3 double knockout mouse in part because of the chromosomal proximity of these two genes which essentially precludes recombination events in a genetic cross between the *Oat1KO* and *Oat3KO*. While probenecid does not exclusively inhibit OAT1 and OAT3 *in vitro*, by treating the *Oat3KO* with this drug, at least for organic anion transport across the basolateral membrane of the renal PT cell, a “chemical double knockout” was essentially created. Since, in the *Oat3KO*, the basolateral transport of organic anions by PT cells is dependent upon the remaining OAT (i.e., OAT1), treatment with probenecid should inhibit basolateral organic anion uptake mediated by OAT1 and lead to the accumulation of these molecules in the plasma, potentially allowing for identification of the uremic toxins with which the transporters interact.

**Table 2:** Uremic Toxins/Retention Solutes Accumulating in the Plasma of Chemical Double Knockout (*Oat3KO* Treated with Probenecid versus Untreated *Oat3KO*).

Metabolite	HMDB ID	KEGG ID	Fold Change (up in plasma)	P-value
Xanthurenate	HMDB00881	C02470	4.96	p ≤ 0.05
Kynurenate	HMDB00715	C01717	4.85	p ≤ 0.05
Xanthosine	HMDB00299	C01762	4.11	p ≤ 0.05
S-adenosylhomocysteine	HMDB00939	C00021	3.57	p ≤ 0.05
Homovanillate sulfate	HMDB11719		3.54	p ≤ 0.05
Isovalerylglycine	HMDB00678		3.25	p ≤ 0.05
4-Hydroxyhippurate	HMDB13678		2.83	p ≤ 0.05
Kynurenine	HMDB00684	C00328	2.25	p ≤ 0.05
Putrescine	HMDB01414	C00134	2.04	p ≤ 0.05
Spermidine	HMDB01257	C00315	1.85	p ≤ 0.05
5-methylthioadenosine	HMDB01173	C00170	1.80	p ≤ 0.05
Cysteine	HMDB00574	C00097	1.71	p ≤ 0.05
Hypoxanthine	HMDB00157	C00262	1.70	p ≤ 0.05
Orotate	HMDB00226	C00295	1.70	p ≤ 0.05
p-Hydroxy-phenyllactate	HMDB00755	C03672	1.50	p ≤ 0.05
Urate	HMDB00289	C00366	1.40	p ≤ 0.05
Threonate	HMDB00943	C01620	1.37	p ≤ 0.05
Dimethylglycine	HMDB00092	C01026	1.30	p ≤ 0.05
Creatinine	HMDB00562	C00791	1.26	p ≤ 0.05
p-Cresol sulfate	HMDB11635	C01468	3.80	0.05 < p < 0.10

Fold Change in plasma concentration of uremic toxins/retention solutes [(*Oat3KO* + probenecid) vs (*Oat3KO* untreated control)].

**Table 3:** Uremic Toxins and/or Retention Solutes Interactions with Oat1KO, Oat3KO, or Chemical Double Knockout.

Metabolite	*In Vivo* Metabolomics			*Existence of *In Vitro* Support for Interaction with Transporter	
	*Oat1KO* Compared to Wildtype	*Oat3KO* Compared to Wildtype	Chemical Double KO (Oat3KO + Probenecid) Compared to *Oat3KO*	OAT1	OAT3
N6-Methyladenosine	Down in urine (A)	—	—		
Phenylsulfate	Up in plasma (A)	—	—		
N2,N2-dimethyl-guanosine	Down in urine (A)	—	—		
Methionine	Up in plasma (A)	—	—		
Xanthurenate	Down in urine (A)	—	Up in plasma (this study)	✓	✓
Orotate	Down in urine (A, B)	—	Up in plasma (this study)		
p-Hydroxy-phenyllactate	Up in plasma (A)	—	Up in plasma (this study)		
S-Adenosylhomocysteine	Up in plasma; this study	—	Up in plasma (this study)		
Cysteine	Up in plasma; this study	—	Up in plasma (this study)	✓	✓
Kynurenate	Up in plasma; this study	—	Up in plasma (this study)	✓	✓
Uric acid	Down in urine (A) Decreased renal secretion (C)	Down in urine (B)	Up in plasma (this study)	✓	✓
Creatinine	Decreased renal secretion (D)	Decreased renal secretion (D)	Up in plasma (this study)	✓	✓
Kynurenine	Up in plasma (A)	Up in plasma (this study)	Up in plasma (this study)	✓	
Indolelactate	Up in plasma (A)	Up in plasma (this study)	—		
Indoxyl sulfate	Up in plasma (A)	Up in plasma (this study)	—	✓	✓
2-Aminophenol sulfate	—	Up in plasma (this study)	—		
2-Oxindole-3-acetate	—	Up in plasma (this study)	—		
Catechol sulfate	—	Up in plasma (this study)	—		
N-Acetyltryptophan	—	Up in plasma (this study)	—		
Mannitol/Sorbitol	—	Up in plasma (this study)	—		
Trimethylamine-N-oxide	—	Up in plasma (this study)	—		
1-Methylimidazoleacetate	—	Up in plasma (this study)	—		✓
Citrulline	—	Up in plasma (this study)	—	✓	
3-carboxy-4-methyl-5-propyl-2-furanpropionate	—	Up in plasma (this study)	—	✓	✓
Imidazole propionate	—	Up in plasma (this study)	—		
Indoleacetate	—	Up in plasma (this study)	—	✓	✓
p-Cresol sulfate	—	Up in plasma (this study)	Up in plasma (this study)	✓	✓
Putrescine	—	Up in plasma (this study)	Up in plasma (this study)		
4-Hydroxyhippurate	—	—	Up in plasma (this study)		
5-Methylthioadenosine	—	—	Up in plasma (this study)		
Dimethylglycine	—	—	Up in plasma (this study)		
Homovanillate sulfate	—	—	Up in plasma (this study)		
Hypoxanthine	—	—	Up in plasma (this study)	✓	✓
Isovaleryglycine	—	—	Up in plasma (this study)		
Spermidine	—	—	Up in plasma (this study)	✓	
Threonate	—	—	Up in plasma (this study)		
Xanthosine	—	—	Up in plasma (this study)		

(A−D) Columns 2–3 indicate findings from previous studies: A–PMID: 2147660, B–PMID:16354673; C – PMID: 18270321; D–PMID: 22338083. (−) Columns 2–4 indicate no significant change detected in plasma concentration of the indicated metabolite in the *in vivo* metabolomics analyses. Blanks in Columns 5–6 indicate that *in vitro* uptake/inhibition studies have not been performed for the indicated metabolite.

*See Supplemental Table 2 for Km, Ki, IC50 and PMIDs.

**Figure 1:**
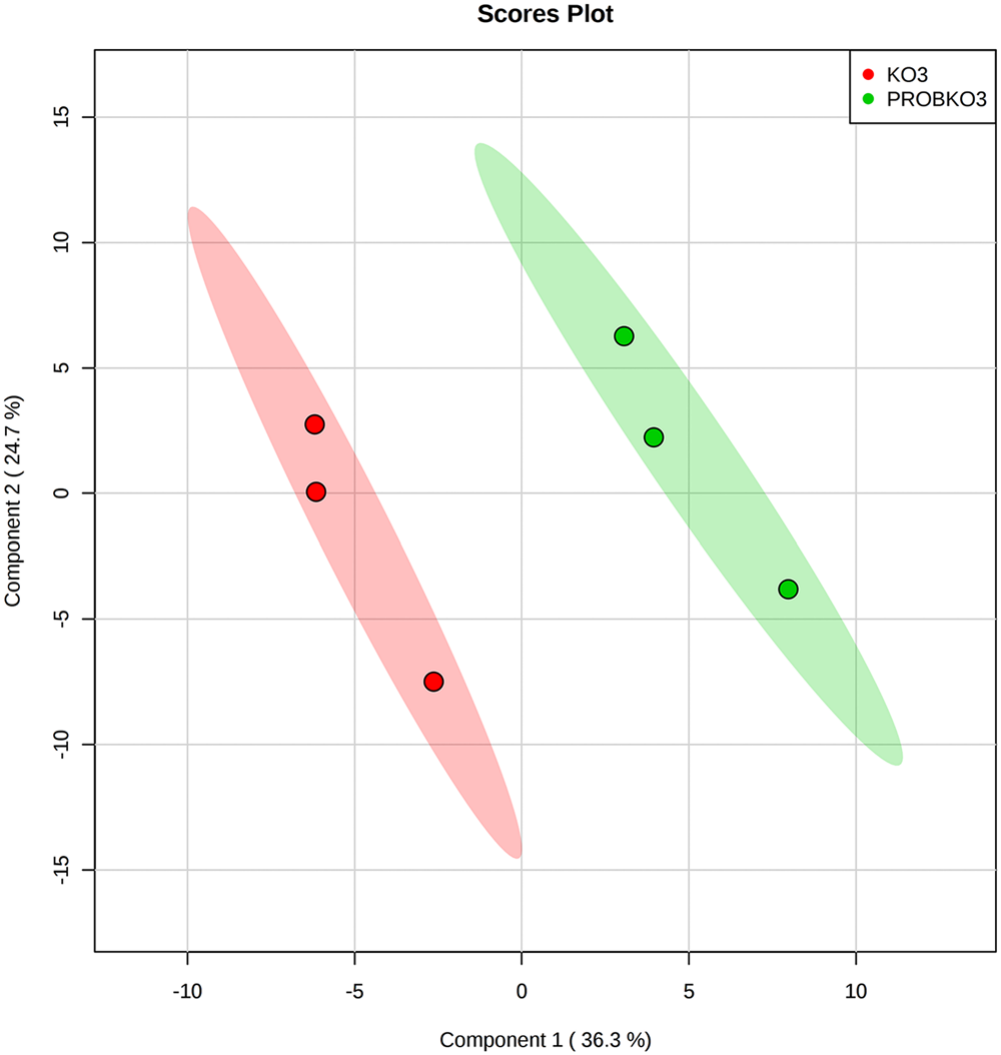
Partial least squares discriminant analysis of uremic toxins/retention solutes reveals separation between plasma metabolites from *Oat3KO* mice (KO3; red) and *Oat3KO* mice treated with probenecid (PROBKO3; green). The dots represent each individual uremic toxin/retention solute metabolite profile and the shaded areas represent the 95% confidence interval.

**Figure 2:**
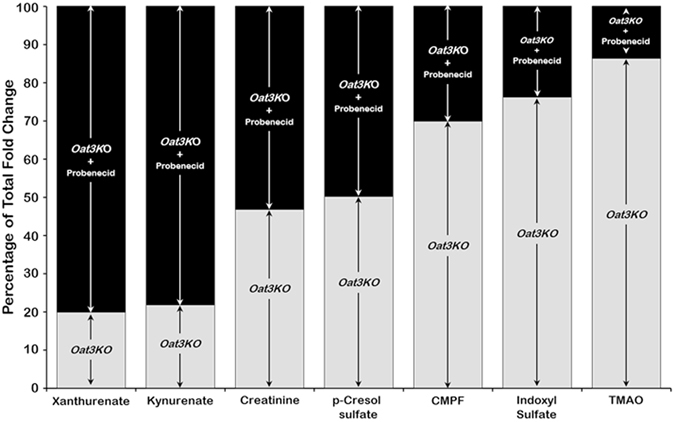
Stacked bar graph of the overall fold change in the plasma levels of the *Oat3KO* for some uremic toxins [i.e., Xanthurenate, Kynurenate, Creatinine, p-Cresol sulfate, 3-carboxy-4-methyl-5-propyl-2-furanpropionate (CMPF), Indoxyl sulfate and Trimethylamine N-oxide (TMAO)]. The data represents the percentage of the total fold change in the overall plasma concentration for each of these toxins/retention solutes in the *Oat3KO* (grey boxes) and in the *Oat3KO* treated with probenecid (black boxes). As described in the text, since in the *Oat3KO*, treatment with probenecid inhibits OAT1-mediated renal uptake, this allows one to see the contribution of each organic anion transporter to the handling of these metabolites (please also see Table 3).

As above, partial least squares discriminant analysis of uremic toxins/retention solutes revealed separation between the *Oat3KO* and probenecid-treated*-Oat3KO* groups based on the metabolite profile of the uremic toxins/retention solutes (Fig. 1) and analysis of the plasma samples derived from these “chemical double knockout” models revealed profound plasma accumulation of several known and/or suspected uremic toxins/solutes compared to the untreated *Oat3KO* (Table 2). For example, kynurenate was only slightly elevated in the plasma of *Oat3KO* mice, however, following treatment of the *Oat3KO* with probenecid, the plasma level of this uremic toxin significantly increased almost 5-fold compared to that seen in the *Oat3KO* (Table 2). This accounted for nearly 80% of the total fold-increase in the plasma seen in the *Oat3KO* (i.e., total of fold-increase seen in both the *Oat3KO* and the probenecid-treated *Oat3KO*). Thus, since, as described above, probenecid is inhibiting OAT1-mediated basolateral uptake from the plasma in the proximal tubule of the *Oat3KO*, this data strongly suggests that the *in vivo* PT uptake and handling of kynurenate is largely mediated by OAT1 (Fig. 2). A similar phenomenon was seen with other toxins/retention solutes, including cysteine, S-adenosylhomocysteine, kynurenine, putrescine, spermidine, orotate and hypoxanthine (Tables 1–3). On the other hand, p-cresol sulfate appears to be handled more or less equally by both OAT1 and OAT3 (Fig. 2), with ~50% of the total fold increase in the plasma of the *Oat3KO* following treatment with probenecid, a finding that is in agreement with *in vitro* data (Supplemental Table 2). In contrast, while 3-carboxy-4-methyl-5-propyl-2-furanpropanoate (CMPF) and trimethylamine-N-oxide (TMAO) were both elevated in the plasma of *Oat3KO* mice, treatment of the *Oat3KO* with probenecid led to relatively small additional accumulations of these metabolites compared to the *Oat3KO* which were not statistically significant, indicating that the majority of the observed plasma accumulation of these metabolites in the *Oat3KO* is likely due to the absence of OAT3 (Fig. 2). This suggests that CMPF and TMAO are more likely to interact with OAT3 than OAT1.

While data supports the notion that CMPF is more a substrate of OAT3 rather than OAT1^25, 32^, recently published *in vitro* data seems to suggest that OAT3 does not directly transport TMAO (albeit this assay was performed at a single high concentration)^25^. This would seem to be consistent with TMAO’s more cationic character; however, an early physiological study found that the renal excretion of TMAO could be inhibited by probenecid, a finding which was, at that time, attributed to probenecid-mediated blockage of the movement of TMAO into the urine via organic anion transporters localized to apical membrane of PT cells^33^. Nevertheless, the finding here of increased plasma levels of TMAO in the *Oat3KO*s would appear to indicate that renal uptake of endogenous TMAO from the plasma is, directly or indirectly, mediated via this basolateral organic anion transporter. It is important to note that OAT3 has been shown to have a greater capacity than OAT1 to interact with cationic compounds, possibly including TMAO^34, 35^.

We have analyzed the *in vivo* roles of renal proximal tubule organic anion transporters, OAT1 and OAT3, individually and in combination, in mediating the uptake and excretion of uremic toxins and uremic retention solutes. Considerable new metabolomics data from the *Oat1KO*, *Oat3KO* and a “chemical double knockout” (*Oat3KO* treated with probenecid) when analyzed in the context of limited previous data clearly links the OATs to the handling of numerous uremic toxins/retention solutes. When considered in light of existing *in vitro* uremic toxin interaction data for these transporters (Supplemental Table 2), it is clear that OAT1 and OAT3 are required for effective renal elimination of many of the “best known” uremic toxins and retention solutes (Table 3).

## Discussion

OAT1/SLC22A6 and OAT3/SLC22A8 are not only both expressed in the basolateral membrane of renal PT cells, but they share a high degree of sequence similarity. In fact, they are each other’s closest homolog and share a great deal of similar functionality^35, 36^. Nevertheless, the data from OAT knockout mice suggests that, while these transporters handle many uremic toxins, certain toxins/solutes prefer OAT1 (e.g., kynurenate, kynurenine, orotate), while others prefer OAT3 (e.g., TMAO, CMPF) with some appearing to be capable of interacting more or less equally with both (e.g., p-cresol sulfate) (Fig. 2; Tables 1–3). Therefore it was of interest to determine if there is an additive or synergistic effect of simultaneously blocking transport mediated by OAT1 and OAT3. Thus, in this study, a “chemical double” knockout was generated by treating the *Oat3KO* with probenecid.

For the majority of the metabolites altered in the *Oat3KO*, it seems that the additional inhibition of OAT1 by probenecid did not significantly alter the plasma levels of the toxin/solute, suggesting that, while the transporters might share some ligands, for the large part the transporters appear to have a significant level of substrate selectivity. Nevertheless, there were a group of toxins/solutes which only show a significant level of accumulation in the plasma when both transporters were inhibited (Table 3), suggesting that both of the transporters contribute to the uptake and elimination of these molecules.

Although we cannot absolutely exclude minor effects of probenecid on other transporters, such as URAT1 (RST) and MRPs, based on available data it seems likely that OAT1 is likely to be the major transporter affected by treatment of the *Oat3KO* with probenecid. For example, probenecid exhibits a significant inhibition potency towards OAT1 (and OAT3) with *K*_i_s in the low micromolar range (i.e., 1.3–32 µM depending on the substrates and model system used), while the inhibition potency towards other transporters has been found to be much higher^37^. For example, the *K*_i_ for OAT2 is 766 µM and MRP4 is >1000 µM, while that for the apical uptake transporter, OAT4, is ~55 µM and for the apical extrusion transporter, MRP2, it is ~45 µM^37^.

Furthermore, early pharmacokinetic studies in rodents demonstrated that the effective unbound plasma concentration of probenecid following a single intraperitoneal dose of probenecid at a concentration of 250 mg/kg was roughly equivalent to that of a human receiving a typical oral dose of 0.5–2.0 g of probenecid^38^, which is in the range of 3–50 µM^39^. This concentration is well within the range of that needed to inhibit OAT1 in the *Oat3KO*, but well below that needed to inhibit OAT2 or MRP4. Although it approaches micromolar concentrations for inhibiting OAT4 and MRP2, these transporters are found on the apical membrane of the proximal tubule cell and are involved in reabsorption from the tubular lumen. Since the endogenous metabolites we are discussing are those which are accumulating in the plasma of the *Oat3KO* treated with probenecid, the inhibition of these transporters is likely to be less of a concern.

Moreover, the likelihood that the changes we are observing are due to probenecid-mediated inhibition of organic anion transporters other than OAT1, such as URAT1 (RST) seems low since metabolomics studies of the knockout and extensive *in vitro* studies of URAT1 (RST) in relation to uremic toxins do not support the view that it transports any uremic toxin other than uric acid^4, 40^, and the many *in vivo* studies of OAT1 and OAT3 knockouts are quite consistent with *in vivo* probenecid data. Moreover, if basolateral OAT1 and OAT3 are blocked by a combination of gene deficiency and probenecid, apical extrusion transporters such as MRPs would not significantly come into play for organic anion uremic toxins in the blood.

Since both of these transporters mediate the pharmacokinetics of a wide variety of drugs, the data raises the possibility that drugs that compete for transport via OAT1 and OAT3 with the uremic toxins have the potential to induce alterations in the levels of the toxins^41^. For example, the accumulation of neurotransmitter metabolites and drugs in the brain in the setting of chronic kidney failure has been suggested to be due to reduced OAT3-mediated brain-to-blood transport due to the increased concentration of uremic toxins such as indoxyl sulfate^42^. Taken together, this could provide a partial explanation for some of the off-target effects of some drugs, including those associated with metabolic syndrome, particularly in the setting of chronic disease and long-term drug treatment^5, 8, 9^. For example, the furan fatty acid metabolite 3-carboxy-4-methyl-5-propyl-2-furanpropanoic acid (CMPF) is a uremic toxin whose renal elimination is mediated by the OATs. This metabolite has recently been shown to act on pancreatic β-cells leading to reduced insulin biosynthesis and represents a potential link to glucose intolerance^32^. Thus, one can see how competition for OAT-mediated clearance of CMPF and a drug could result in a drug-metabolite interaction at the level of the renal transporter affecting the concentrations, not only of the drug, but of the endogenous metabolite as well, which in the case of CMPF could lead to alterations in glucose tolerance with its effects on the pancreas.

In further support of this notion, it was recently demonstrated that administration of either ketoprofen or diclofenac (non-steroidal anti-inflammatory drugs (NSAIDs) capable of inhibiting OAT1- and OAT3-mediated transport), significantly decreased the renal clearance of indoxyl sulfate thereby increasing systemic exposure to this uremic toxin^43^. It was suggested that this NSAID-mediated increase in the concentration of indoxyl sulfate, might contribute to the progression of indoxyl sulfate-induced cardiovascular disease and, at least in part, explain the pathogenesis of analgesic nephropathy^44^. Taken together, the major point is that partial blockade of OAT1- or OAT3-mediated transport of uremic toxins by competing drugs has the potential to alter the concentrations of certain toxins and lead to the distal cascade effects of the toxin.

Our study also supports the relevance of the remote sensing and signaling hypothesis to the pathophysiology of uremia and CKD^5, 8, 9, 45–48^. This hypothesis emphasizes the centrality of multispecific SLC and ABC transporters to inter-organ and inter-organismal small molecule communication throughout the body and thus the importance of the transporters to modulation of metabolism and signaling^5, 8, 9, 30, 45, 47, 49, 50^. In this regard, it is worth noting that some of the uremic toxins are derived from metabolites generated by the microbiome in the gut. For example, indoxyl sulfate, ultimately derived from metabolism of tryptophan by the gut bacteria, is absorbed from the intestine into the blood as indole, which is then sulfated in the liver to indoxyl sulfate which is transported into various organs and/or excreted by the kidney^51^. This amounts to interorganismal movement of a small organic anion capable of inducing toxicity or activating signaling pathways via nuclear receptor signaling. For example, indoxyl sulfate, kynurenine and kynurenate signal via the aryl hydrocarbon receptor (AHR)^52–54^, which is expressed in most tissues, including those affected in uremia such as the liver, intestine and kidney^55, 56^. Kynurenate can also activate the orphan G-couple protein receptor, GPR35^57^, which is largely expressed in tissues affected in uremia, such as the gut and CNS^58, 59^. CMPF, on the other hand, has been implicated in a glucose sensing mechanism involving OAT3, which is expressed in the pancreas (among other non-renal tissues), leading to altered metabolism^32^. Of note, metabolic reconstructions of “omics” data from the *Oat1KO* implicated regulation by OAT1 and OAT3 of a number of biochemical pathways reported to be altered in uremia (e.g., polyamine and purine metabolism)^11^. Although much more work needs to be done in this regard, it is possible that high levels of uremic toxins inhibit the normal metabolic functions of the OATs via uremic toxin-metabolite competition at the level of the transporter; certainly there is evidence for this in the case of uremic toxin-drug competition^25, 43^.

The results support the roles of organic anion transporters in the handling of a number of uremic toxins/retention solutes that follow interorgan and interorganismal (gut microbiome-host) communication pathways that may include the activation of key signaling pathways affecting a wide variety of metabolic processes, including those which are known to be affected in uremic syndrome pathophysiology^46^. This general view is also supported by metabolic reconstructions of “omics” data from the *Oat1KO* and *Oat3KO* mice^11, 30, 49^, where it has been demonstrated that Oats regulate biochemical pathways of interorgan and interorganismal communication such as those involving gut microbiome metabolites, energy metabolism, purine metabolism, antioxidants and lipid metabolism and many other pathways altered in CKD. Taken together, this suggests that uremia is in part a disease of disordered remote sensing and signaling^5, 8, 9, 11, 45–47^.

## Methods

### Animals

All experimental protocols were approved by The University of California San Diego Institutional Animal Care and Use Committee (IACUC). The animals were handled in accordance with the Institutional Guidelines on the Use of Live Animals for Research; all experiments involving the use of animals were also conducted in accordance with the Institutional Guidelines on the Use of Live Animals for Research. Adult (n = 3) wildtype, *Oat1*- and *Oat3-*deficient male mice received a single, daily intraperitoneal (i.p.) injection of either 200 mg/kg water-soluble probenecid (Invitrogen, Carlsbad, CA) [0.02 mg/µL in PBS (10 µL/g of body weight)] or PBS (sham-treated control) for three days. The animals were housed separately under a 12-h light-dark cycle and were provided access to food (standard diet) and water *ad libitum*. On day three, two hours before samples were taken, the mice were given a final i.p. injection of either probenecid or PBS. Two hours after this last treatment, blood was collected, and plasma was isolated and stored at −80 °C until being shipped for analysis as described below.

### Metabolomic Analysis, Data Extraction, Compound Identification, Curation and Statistics

Individual, unpooled samples were measured by the Metabolon analytical system (Metabolon, Inc., Durham, NC)^60, 61^. Samples were prepared and subjected to ultrahigh performance liquid chromatography-tandem mass spectroscopy (UPLC-MS/MS) utilizing an ACQUITY ultra-performance liquid chromatography (UPLC) (Waters, Milford, MA) and a Q-Exactive high resolution/accurate mass spectrometer interfaced with a heated electrospray ionization (HESI-II) source and Orbitrap mass analyzer operated at 35,000 mass resolution (Thermo Scientific, Waltham, MA). Raw data was extracted, peak-identified and QC processed using Metabolon’s hardware and software^60, 61^. Two-way ANOVA testing was used to calculate the p-values and the metabolites that were selected for display in figures and tables had either: (1) a fold change ≥1.2 with a p-value ≤ 0.05; or (2) a fold change ≥2.0 with a p-value ≤ 0.1 in at least one of the various comparisons (e.g., *Oat1KO* vs WT; *Oat3KO* vs WT; *Oat3KO* + probenecid vs *Oat3KO*). The separability of the uremic toxins/retention solutes in the wildtype, *Oat3KO*, and probenecid-treated *Oat3KO* plasma samples was assessed by partial least squares discriminant analysis (PLS-DA) using Metaboanalyst 3.0 (http://www.metaboanalyst.ca/)^62^.

## Electronic supplementary material

Supplemental Data
