# Supplementary material for: Key Role for the Organic Anion Transporters, OAT1 and OAT3, in the in vivo Handling of Uremic Toxins and Solutes
Source: Sci Rep. 2017 Jul 10;7:4939. doi: 10.1038/s41598-017-04949-2 (PMC5504054; doi:10.1038/s41598-017-04949-2)
Supplement: Supplementary file 1 — Supplemental Data [file 41598_2017_4949_MOESM1_ESM.pdf]

Key Role of the organic anion transporters, OAT1 and OAT3, in the in vivo Handling of Uremic Toxins and Solutes

Wei Wu, Kevin T. Bush and Sanjay K. Nigam

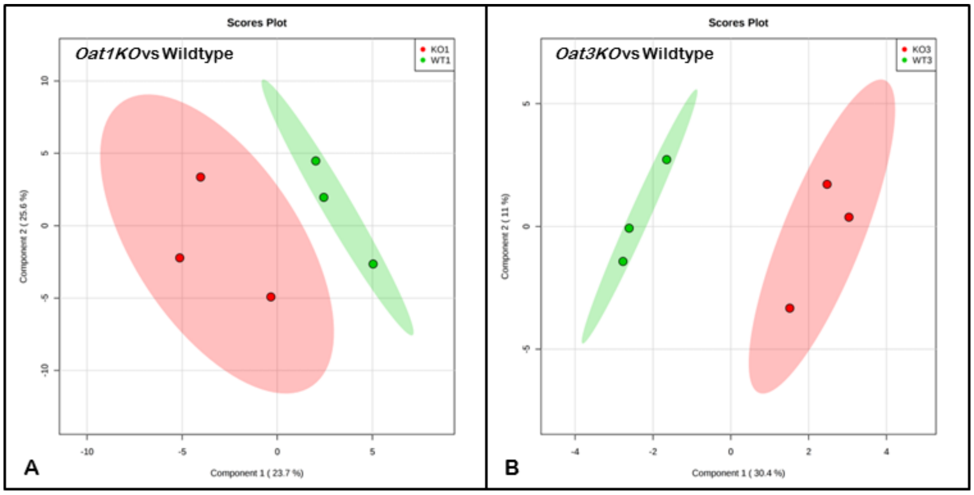

Supplemental Figure 1. Partial least squares discrimination analysis reveals separation between plasma metabolites from (A) Oat1KO (KO1; red) and wild-type (WT1; green) mice and (B) Oat3KO (KO3; red) and wildtype (WT3; green) mice. The dots represent each individual metabolite profile and the shaded areas represent the 95% confidence interval**.**

SUPPLEMENTAL TABLE S1<br>List of Water-soluble and Protein-bound<br>Uremic Toxins/Retention Solutes			
Metabolite	HMDB	KEGG	Metabolon
1,3,7-Trimethyluric acid	HMDB02123	-	
1,7-Dimethyluric acid	HMDB11103	-	
1-Methylguanosine	HMDB01563	C04545	
1-Methylhistidine	HMBD00001	C01152	30460
1-Methylinosine	HMDB02721	-	
1-Methyluric acid	HMDB03099	C16359	
2,5-Furandicarboxylic acid	HMDB04812	-	
2-Aminophenol sulphate	HMDB61116	-	43266
2-Furoylglycine	HMDB00439	-	
2-Heptenal	HMDB33827	-	
2-Hexenal	HMDB31496	C08497	
2-Methoxyphenol sulfate			
2-Nonenal	HMDB31269	-	
2-Octenal	HMDB30961	-	
2-Oxindole-3-acetate			40479
3-Aminoisobutanoic acid	HMDB03911	C05145	
3-carboxy-4-methyl-5-propyl-2-furanpropanoate	HMDB61112		31787
3-Deoxyglucosone			
3-Hydroxyhippuric acid	HMDB06116	-	
3-indoxyl sulfate	HMDB00682		27672
3-Methylcatechol sulfate			46165
3-Methylglutarylcarnitine	HMDB00552	-	
3-Methylhistidine	HMDB00479	C01152	15677
4-Acetamidobutanoic acid	HMDB03681	C02946	1558
4-Decenal	HMDB41014	-	
4-guanidinobutanoate	HMDB03464	C01035	15681
4-HO-Decenal			
4-HO-Hexenal			
4-HO-Nonenal			
4-HO-Octenal			
4-Hydroxyhippuric acid	HMDB13678	-	33959
4-Methylcatechol sulfate			46146
4-Pyridoxic acid	HMDB00017	C00847	
4-Vinylphenol sulfate			
5'-Methylthioadenosine	HMDB01173	C00170	1419
6-Sialyl-N-acetyllactosamine	HMDB06584	-	
7-Methyluric acid	HMDB11107	C16355	
8-Hydroxy-2’-deoxyguanosine	HMDB03333		
Acetylcarnosine	HMDB12881	-	43488
Acisoga			
Allantoin	HMDB00462	C01551	1107
Alpha-CEHC glucuronide			
Alpha-N-Phenylacetyl-L-glutamine	HMDB06344	C04148	
Androsterone sulfate	HMDB02759	C04555	
Angiogenin			
Anthranilate	HMDB01123	C00108	4970
Arab(in)itol	HMDB01851	C00532	
Arabinonic acid	HMDB00539	C00878	
Arabitol §	HMDB01851	C00532	
Argininic Acid	HMDB03148	-	
Asymetric Dimethylarginine	HMDB01539	C03626	36808
Beta-Alanine	HMDB00056	C00099	
Cinnamoylglycine	HMDB11621	-	38637
Citrulline	HMDB00904	C00327	2132
C-mannosyltryptophan			
Creatine	HMDB00064	C00300	27718
Creatinine	HMDB00562	C00791	513
Cysteine	HMDB00574	C00097	1868
Cytidine	HMDB00089	C00475	514
Decanal	HMDB11623	C12307	
Dihydroxyphenylalanine	HMDB00181	C00355	
Dimethylamine	HMDB00087	C00543	
Dimethylglycine	HMDB00092	C01026	5086
D-Threitol	HMDB04136	C16884	
D-Xylose	HMDB00098	C00181	

Erythritol	HMDB02994	C00503	20699
Erythronic acid	HMDB00613	-	42420
Ethylamine	HMDB13231	C00797	
Fructoselysine	HMDB34879		
Fumaric acid	HMDB00134	C00122	1643
Galactitol	HMDB00107	C01697	
Gamma-CEHC	HMDB01931	-	
Gamma-CEHC glucuronide			
Gluconic acid	HMDB00625	C00257	587
Glutarylcarnitine	HMDB13130	-	
Glycine	HMDB00123	C00037	58
Glyoxal			
Guanidine	HMDB01842	C17349	
Guanidinosuccinic acid	HMDB03157	C03139	
Heptanal	HMDB31475	C14390	
Hexanal	HMDB05994	C02373	
Hippurate	HMDB00714	C01586	15753
Homocitrulline	HMDB00679	C02427	22138
Homocysteine	HMDB00742	C05330	
Homovanillic acid sulfate	HMDB11719	C05582	38349
Hypoxanthine	HMDB00157	C00262	3127
Imidazolepropionic acid	HMDB02271	-	40730
Indican		-	
Indole-3-methyl acetate	HMDB29738	-	
Indoleacetate	HMDB00197	C00954	27513
Indoleacetyl glutamine	HMDB13240	-	35527
Indolelactic acid	HMDB00671	C02043	18349
Indoxyl-β-D-glucoronide	HMDB10319	C03033	
Inosine	HMDB00195	C00294	1123
Insulin-like growth factor 1			
Interleukin-10			
Isobutyrylglycine	HMDB00730	-	
Isobutyryl-L-carnitine	HMDB00736	-	33441
Isovalerylglycine	HMDB00678	-	35107
Kynurenate	HMDB00715	C01717	1417
Kynurenine	HMDB00684	C00328	15140
L-Arabinose	HMDB00646	C00259	
Leptin			
Levoglucosan	HMDB00640	-	
Levoinositol	HMDB34220	-	
L-Fucose	HMDB00174	C01019	
L-gamma-glutamyl-L-isoleucine	HMDB11170	-	
L-Gulonolactone	HMDB03466	C01040	
L-Xylonate	HMDB60256	C05411	
Malondialdehyde	HMDB06112	C19440	
Mannitol	HMDB00765	C00392	46142
Melatonin	HMDB01389	C01598	
Methionine	HMDB00696	C00073	1302
Methylglyoxal	HMDB01167	C00546	
Methylguanidine	HMDB01522	C02294	
Methylimidazoleacetic acid	HMDB02820	C05828	32350
Monomethylamine	HMDB00164	C00218	
myo-inositol	HMDB00211	C00137	1124
N1-Methyl-2-pyridone-5-carboxamide	HMDB04193	C05842	40469
N1-methyladenosine	HMDB03331	C02494	15650
N2,N2-Dimethylguanosine	HMDB04824		
N2,N5-diacetylornithine			
N4-Acetylcytidine	HMDB05923		
N6-carbamoyl-threonyl-adenosine	HMDB41623		35157
N6-carboxymethyllysine			36713
N6-Methyladenosine	HMDB04044	-	
N-Acetyl alliin			
N-acetyl-1-methylhistidine			43255
N-acetyl-3-methylhistidine			43256
N-acetylarginine	HMDB04620	C02562	33953
N-Acetylhistidine	HMDB32055	C02997	33946
N-Acetyl-L-alanine	HMDB00766	C00624	1585
N-Acetyl-L-methionine	HMDB11745	C02712	1589
N-Acetyl-L-phenylalanine	HMDB00512	C03519	33950
N-Acetylneuraminic acid	HMDB00230	C19910	32377
N-Acetylproline			
N-Acetylserine	HMDB02931	-	37076
N-Acetylthreonine			33939
N-Acetyltryptophan	HMDB13713		33959

N-Acetylvaline	HMDB11757	-	1591
Neopterin	HMDB00845	C05926	
N-Formyl-L-methionine	HMDB01015	C03145	2829
Nicotinamide	HMDB01406	C00153	594
N-methyl-4-pyridone-3-carboxamide	HMDB04194	C05843	
Nonanal	HMDB59835		
Orotate	HMDB00226	C00295	1505
Orotidine	HMDB00788	C01103	35172
Osteocalcin			
O-sulfo-L-tyrosine			45413
Oxalate (ethanedioate)	HMDB02329	C00209	20694
Pantothenic acid	HMDB00210	C00864	
p-Cresol sulfate	HMDB11635	C01468	36103
Pentosidine	HMDB03933		
Phenol	HMDB00228	C00146	
Phenol sulphate	HMDB60015	C00850	32553
Phenylacetic acid	HMDB00209	C07086	
Phenylacetylglutamine	HMDB06344	C04148	
Phenylacetylglycine	HMDB00821	C05598	33945
Phenylcarnitine			
p-Hydroxyphenylacetic acid	HMDB00755	C03672	32197
p-Hydroxyphenylacetic acid	HMDB00020	C00642	
Proline betaine	HMDB04827	C10172	34384
Prolylhydroxyproline	HMDB06695	-	35127
Pseudouridine	HMDB00767	C02067	33442
Putrescine	HMDB01414	C00134	1408
Pyrocatechol sulfate	HMDB59724	-	35157
Pyroglutamylvaline			
Quinolinate	HMDB00232	C03722	1899
Retinol Binding Protein			
Riboflavin	HMDB00244	C00255	
Saccharin	HMDB29723	C12283	
S-adenosyl-homocysteine	HMDB00939	C00021	42382
Salicyluric acid	HMDB00840	C07588	18281
Salicyluric glucuronide			
Scyllitol	HMDB06088	C06153	
Sorbitol	HMDB00247	C00794	46142
Spermidine	HMDB01257	C00315	485
Sucrose	HMDB00258	C00089	
Symetric Dimethylarginine	HMDB03334	C03626	36808
Tartaric acid	HMDB00956	C00898	
Taurocyamine	HMDB03584	C01959	
Threitol	HMDB04136	C16884	
Threonic acid	HMDB00943	C01620	27738
Tiglylglycine	HMDB00959	-	
Trimethylamine	HMDB00906	C00565	
Trimethylamine N-oxide	HMDB00925	C01104	40406
Urate	HMDB00289	C00366	1604
Urea	HMDB00294	C00086	1670
Uridine	HMDB00296	C00299	606
Vanillic acid	HMDB00484	C06672	
Vanillylmandelic acid	HMDB00291	C05584	
Vascular endothelial growth factor			
Xanthine	HMDB00292	C00385	3147
Xanthosine	HMDB00299	C01762	15136
Xanthurenate	HMDB00881	C02470	15679
α1-Acid glycoprotein			
α-keto-δ-Guanidinovaleric Acid	HMDB04225	C03771	
β-Guanidinopropionic Acid	HMDB13222	C03065	
β-Lipotropin			
PMID: 22626821; 27467266; 22069747; 23941498; 20378825; 26317986;19708975			

SUPPLEMENTAL TABLE S2

Uremic Toxins and/or Retention Solutes for Which In Vitro Data Exists
Indicating Ability to Interact with OAT1 and/or OAT3

Metabolite	HMDB	KEGG	Km (uM)		Ki (uM)		IC50 (uM)		PMID
			OAT1	OAT3	OAT1	OAT3	OAT1	OAT3	
2-Aminobenzoic acid	HMDB01123	C00108			inhibitor	inhibitor			15944205
3-carboxy-4-methyl-5-propyl-2-furan-propionate	HMDB61112	-	85.3<br>141<br>154	18.6<br>26.5<br>10.9<br>6.43	103	27.9	79	4.01<br>28	14675047<br>15846473<br>27467266
Citrulline	HMDB00904	C00327	238<br>373						19403644
Creatinine	HMDB00562	C00791	6.7	>10mM			14000	40000	22338083<br>27467266
Cysteine	HMDB00574	C00097			inhibitor	inhibitor			16164645
Hippurate	HMDB00714	C01586	12.2<br>23.5<br>27.5		27.5<br>18.8<br>55.6	18.6<br>18	20<br>2077<br>31	11.9<br>41	11815391<br>14675047<br>15846473<br>27467266
Hypoxanthine	HMDB00157	C00262			inhibitor	inhibitor			16038872
Indoleacetate	HMDB00197	C00954	23.6<br>14<br>47.1		48.5<br>21	582	83<br>140	509	11815391<br>14675047<br>15846473<br>27467266
Indoxyl sulfate	HMDB00682	-	18<br>32.9<br>20.5<br>17.7	263<br>174	25<br>13.2<br>22.7<br>34.2	138	83<br>50<br>47.3<br>110	270	11815391<br>14675047<br>15846473<br>27467266
Kynurenate	HMDB00715	C01717					34	8<br>23	15944205<br>27467266
Kynurenine	HMDB00684	C00328			1.4		12		21476605
p-Cresol sulfate	HMDB11635	-					210	200	27467266
Quinolinate	HMDB00232	C03722			inhibitor				15944205
Spermidine	HMDB01257	C00315			235		2000		21757732
Spermine	HMDB01256	C00750			188		1600		21757732
Uric acid	HMDB00289	C00366	197.6<br>943	380.3	304	287	312.5<br>2200	290<br>670	16038872<br>17674156<br>27467266
Xanthine	HMDB00292	C00385			238		243.9		16038872
Xanthurenate	HMDB00881	C02470					15	11.5	15944205
